# De novo transcriptome assembly and data for the blue-winged teal (*Spatula discors*)

**DOI:** 10.1016/j.dib.2020.105380

**Published:** 2020-03-05

**Authors:** Amanda C. Dolinski, Jared J. Homola, Mark D. Jankowski, Jennifer C. Owen

**Affiliations:** a Department of Fisheries and Wildlife, Michigan State University, East Lansing, MI, United States; b Department of Large Animal Clinical Sciences, Michigan State University, East Lansing, MI, United States; c U.S. Environmental Protection Agency, Region 10, Seattle, WA 98101,United States

**Keywords:** Avian, Transcriptome, Influenza, Trinity, RNAseq

## Abstract

The blue-winged teal (*Spatula discors*) is a recreationally and ecologically important dabbling duck species in North America. Transcriptomic data of this species can be used in public and animal health studies given its role as a natural reservoir host for avian influenza, which can be a zoonotic disease of high concern. Ileum and bursa of Fabricius tissues were sampled from six captive raised blue-winged teals, four of the six who were experimentally infected with low-pathogenic avian influenza virus H5N9. RNAseq data were generated from extracted total mRNA from each tissue and pooled to create a *de novo* assembly of the transcriptome using Trinity. A total of 571,105 transcripts were identified at 449,956 unique unigenes that have been functionally annotated. This transcriptome will be useful for future blue-winged teal gene expression research, especially in hypothesis driven differential expression studies to determine the driving forces of avian influenza host-pathogen interactions, spatial distribution, and transmission.

**Table:** **Specification Table**

Subject	Biology
Specific Subject Area	Virology and Transcriptomics
Type of data	Transcriptome assembly, raw sequences
How data were acquired	High-throughput RNA sequencing by the University of Minnesota Genomics Center (UMGC) using Illumina Hi-Seq 2500
Data format	Analyzed, raw
Experimental factors	Teal eggs were hatched and raised in captivity. Four birds were inoculated with low-path avian influenza virus, two birds were sham inoculated.
Experimental features	Total RNA was extracted from three ileum and three bursa samples from six birds total. cDNA library preparation and sequencing were performed at UMGC. Bioinformatic analyses were completed by the authors.
Description of data collection	RNAseq data was generated from ileum and bursa tissue, then analyzed to create a *de novo* transcriptome using Trinity.
Data source location	Teal eggs were collected from uncultivated wetlands in Cando, Towner County, North Dakota (48.457, −99.319) and shipped to Michigan State University in East Lansing, Ingham County, Michigan where the eggs were incubated, hatched, and raised in captivity, then experimentally infected with 5.63 log EID_50_ (LPAIV) H5N9 A/northern pintail/California/44,221-761/2006 acquired from USGS National Wildlife Health Center in Madison, Dane County, Michigan.
Data accessibility	The assembled *de novo* transcriptome and associated annotation data are available on Mendeley Data. All analysis scripts are available in a GitHub repository (https://github.com/OwenLabMSU/bwteDeNovo) DOI: 10.5281/zenodo.3716560.

## Value of the Data

• To our knowledge, this is the first published transcriptome assembly for the blue-winged teal. It will be broadly useful as a reference for future blue-winged teal research ranging from breeding and nutrition studies to disease ecology, toxicology, and host-pathogen interaction studies involving the blue-winged teal, specifically those infected with avian influenza virus.
• These data will benefit ornithologists, wetland biologists, nutritionists, ecologists, veterinarians, and epidemiologists who are interested in studying transcriptomic processes in blue-winged teals and other closely related *Anas* species.
• The blue-winged teal transcriptome can be used as a reference for differential expression analysis of RNAseq data.

## 1 Data

**Fig. 1:**
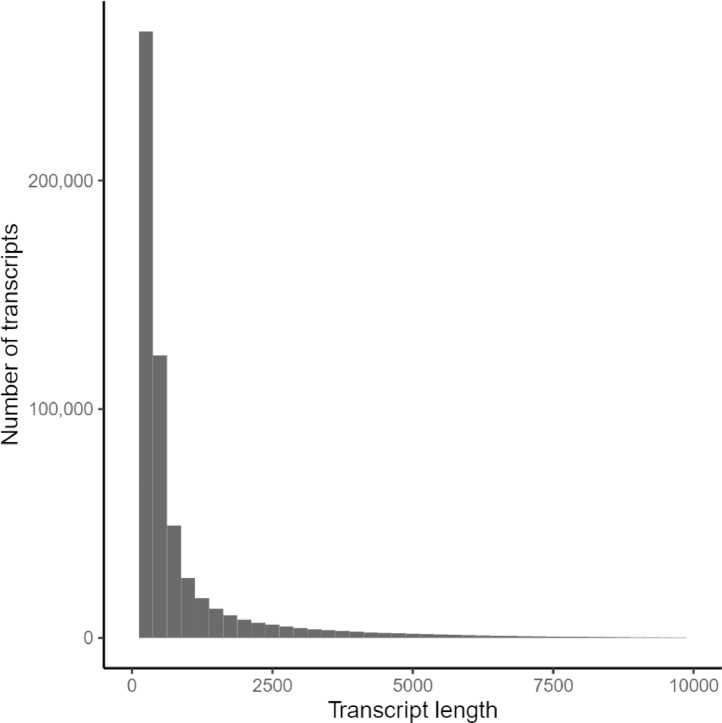
Histogram of transcript lengths in *de novo* blue-winged teal transcriptome.

**Fig. 2:**
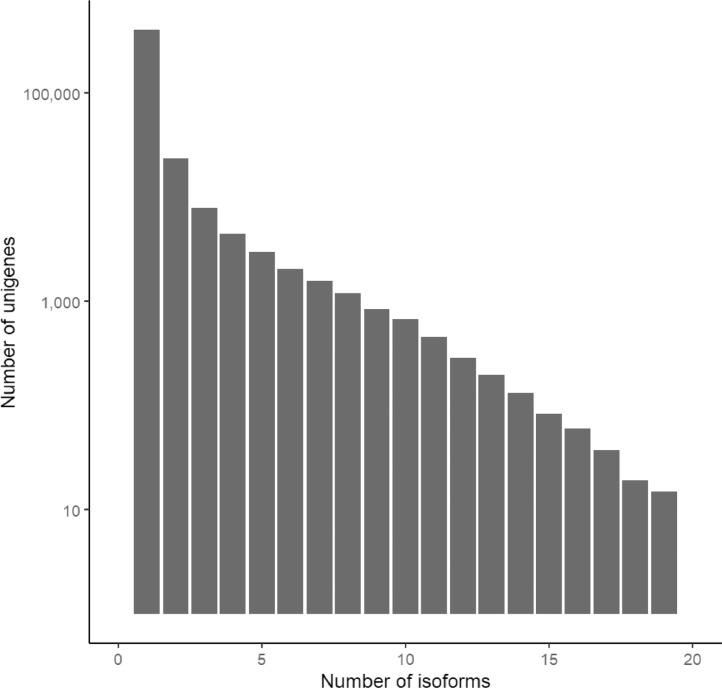
Number of isoforms per Trinity assembled unigene in *de novo* blue-winged teal transcriptome.

Data described in this article originate from cDNA sequencing of two tissue types (Ileum and bursa of Fabricius) from six blue-winged teals (*Spatula discors*) that were subsequently assembled into a *de novo* transcriptome using Trinity [1]. We present quality assessment information on the raw sequencing data and assembled transcriptome, as well as functional annotations derived from several databases. Table 1 describes summary statistics of the transcriptome, Table 2 describes predicted open reading frames, Table 3 provides the number of occurrences for the most common gene ontology (GO) terms, and Table 4 provides counts of the number of transcripts associated with the 15 more frequently occurring Kyoto Encyclopedia of Genes and Genomes (KEGG) identifiers. Fig. 1 shows distribution of transcript lengths and Fig. 2 is a histogram of the number of isoforms per assembled unigene.

**Table 1:** Summary statistics of the *de novo* assembly of the blue-winged teal transcriptome.

		Raw assembly	Final assembly
	Number of unigenes	474,556	449,956
	Number of transcripts	677,825	571,105
	% GC	45.75	45.51
All transcripts	Total assembled bases	705,018,715	528,964,935
	N10	8432	7836
	N30	4665	4139
	N50	2544	2077
	Mean contig length	1040.12	926.21
Longest isoform per unigene	Total assembled bases	305,174,493	295,732,957
	N10	6562	6630
	N30	2280	2375
	N50	903	943
	Mean contig length	643.07	657.25

**Table 2:** Results from TransDecoder describing predicted open reading frames (*n* = 166,115).

Types of coding sequence	N
Complete proteins (including start and stop codons)	83,794
3-prime partial alignments	13,220
5-prime partial alignments	48,319
Internal alignments	20,782

**Table 3:** Number of occurrences for the ten most common gene ontology (GO) terms for each of three primary categories.

	Term	N_GO terms_
Cellular component	Nucleus	38,973
	Cytoplasm	36,855
	Cytosol	32,416
	Plasma membrane	25,064
	Integral component of membrane	22,763
	Nucleoplasm	19,572
	Membrane	12,446
	Mitochondrion	8488
	Integral component of plasma membrane	7749
	Golgi apparatus	6949
Molecular function	Metal ion binding	17,817
	ATP binding	17,225
	DNA binding	9458
	RNA binding	8841
	Zinc ion binding	7384
	Identical protein binding	6821
	Protein homodimerization activity	6045
	Calcium ion binding	5207
	DNA-binding transcription factor activity	4023
	Protein serine/threonine kinase activity	3844
Biological Process	Positive regulation of transcription by RNA polymerase II	6838
	Negative regulation of transcription by RNA polymerase II	5095
	Signal transduction	4574
	Positive regulation of transcription, DNA-templated	4154
	Apoptotic process	3935
	Intracellular signal transduction	3826
	Cell differentiation	3718
	Cell division	3424
	Protein phosphorylation	3424
	Regulation of transcription by RNA polymerase II	3420

**Table 4:** Number of transcripts assigned to each of the 15 most common KEGG identifiers.

KEGG Identifier	N_Transcripts_	Definition
K06712	530	BTN; Butyrophilin
K09228	186	KRAB; KRAB domain-containing zinc finger protein
K01530	169	E7.6.2.1; Phospholipid-translocating ATPase
K00901	148	dgkA, DGK; diacylglycerol kinase (ATP)
K00710	141	GALNT; polypeptide N-acetylgalactosaminyltransferase
K01081	133	E3.1.3.5; 5′-nucleotidase
K10356	130	MYO1; myosin I
K10408	122	DNAH; dynein heavy chain, axonemal
K16449	108	RGS; regulator of G-protein signaling
K19721	103	COL5AS; collagen, type V/XI/XXIV/XXVII, alpha
K20404	102	DEPDC5; DEP domain-containing protein 5
K16342	98	PLA2G4, CPLA2; cytosolic phospholipase A2
K06545	96	CD163; CD163 antigen
K08823	93	CLK2_3; dual specificity protein kinase CLK2/3
K06115	92	SPTB; spectrin beta

## 2 Experimental design, materials, and methods

### 2.1 Birds

Blue-winged teal eggs (*n* = 6) were collected from nests (1 per nest) in uncultivated fields in Cando, North Dakota under U.S. Fish and Wildlife Scientific Collection permit #MB194270 and North Dakota Game and Fish Department License #GNF0363940. Birds were raised in captivity until when they were 9–12 weeks of age.

### 2.2 Virus

LPAIV H5N9 A/northern pintail/California/44,221-761/2006 was acquired from The United States Geological Survey National Wildlife Health Center in Madison, Wisconsin and propagated in pathogen-free chicken eggs (Charles River Laboratories, Wilmington, MA, USA) at Michigan State University. Virus inoculum was diluted in Dulbecco's Modified Eagle Medium (+4.5 g/L D-Glucose, + L-Glutamine; ThermoFisher Scientific, Waltham, MA, USA) to a concentration of 5.63 log EID_50_/ml. Birds were inoculated with virus via nares and esophagus.

### 2.3 Sample collection

Ileum intestinal tissue was collected from three birds (two males and one female), and the Bursa of Fabricius (hereafter ‘bursa’) was collected from the other three (one male and two females). The two females in which the ileum was collected and the two males in which the bursa was collected were experimentally infected with virus and tested positive as detected by RT-PCR targeting the matrix protein gene of viral RNA extracted from cloacal swabs [2]. The other two birds were sham-inoculated and not positive for virus. All tissues were collected one day post inoculation. Tissue samples were placed in RNAlater (Sigma-Aldrich, St. Louis, MO, USA) and stored at room temperature for 24 h, then removed from RNAlater and stored at −80 °C.

### 2.4 RNA extraction, cDNA library construction, and sequencing

Total mRNA was extracted from each tissue using the Qiagen RNeasy kit according to the manufacturer's protocol (Cat #74,104, Qiagen, Inc, Hilden, Germany). RNA RIN scores averaged 9.0 and ranged from 7.4 to 9.8. Each RNA extract had a dual-indexed TruSeq stranded mRNA library created. All libraries were combined into a pool and sequenced across 1.5 lanes of a HiSeq 2500 2 × 125-bp run using v4 chemistry, which generated approximately 320 M pass filter reads for the pool or approximately 53.3 M reads per sample. All expected barcodes were detected and well represented. Mean quality scores for all libraries were ≥ Q30. The libraries were gel size selected to have inserts of approximately 200 bp.

### 2.5 Transcriptome analysis

Quality of raw reads was examined using FastQC (v. 0.11.7) [3]. Reads were quality filtered and had Illumina adapters removed using Trimmomatic (v. 0.38) [4]. Quality filtering was performed by removing bases with a Phred score lower than 15 across a four base sliding window, as well as reads with a length shorter than 40 bp. Next, we filtered for potential rRNA contamination by using Bowtie2 [5] to align all reads to the SILVA small and large rRNA subunit databases [6,7]. A total of 296,155,317 paired reads remained after filtering was completed. Bowtie2 alignments were performed using the –very-sensitive-local configuration and all reads that did not align were retained for further analysis.

### 2.6 De novo assembly and annotation

*De novo* transcriptome assembly was completed using Trinity [1] with default options and requiring a minimum contig length of 200 nt. Clustering of similar transcripts was performed using CD-HIT-EST [8,9] and transcript were quality filtered using TransRate [10]. This filtered transcriptome contained 571,105 transcripts associated with 449,956 unigenes. Quality assessment of read alignments was conducted using Bowtie2 to evaluate our ability to align transcripts to the *de novo* assembly, which provided a 98.5% realignment rate. To assess transcriptome completeness, BUSCO v3 [11,12] was used with the aves ortholog database, identifying 86.8% complete, 7.2% fragmented, and 6.0% missing single copy orthologs. Next, TransDecoder (v. 2.1.0) [13] was used to identify candidate coding regions prior to transcriptome annotation using the Trinotate pipeline (v. 3.2.0) [14]. Alignments to the SwissProt database were performed using blastx for the entire transcriptome and blastp for the subset of putative coding regions using a minimum e-value of 1e-5. Blastx annotations were recovered for 135,042 transcripts and blastp annotations were provided for 75,404 transcripts. Trinotate was also used to perform further functional annotations including GO terms (Table 3) and designations from the KEGG (Kyoto Encyclopedia of Genes and Genomes) [15] and eggNOG [16] databases and for identifying protein domains in PFAM [17]. Finally, we used Kallisto [18] to quantify expression levels within the data used to construct the transcriptome, finding 130,077 transcripts with transcripts per millions (TPM) values >0.05 and 69,074 transcripts with >1.0 TPM.

## References

[1] Grabherr M.G., Brian N., Haas J., Yassour Moran, Levin Joshua Z., Thompson Dawn A., Amit Ido, Adiconis Xian, Fan Lin, Raychowdhury Raktima, Zeng Qiandong, Chen Zehua, Mauceli Evan, Hacohen Nir, Gnirke Andreas, Rhind Nicholas, di Palma Federica, Bruce W., Friedman A.R. (2013). Trinity: reconstructing a full-length transcriptome without a genome from RNA-Seq data. Nat. Biotechnol..

[2] Spackman E., Suarez D.L. (2008). Type A influenza virus detection and quantitation by real-time RT-PCR. Avian Influenza Virus.

[3] S. Andrews, FastQC: A Quality Control Tool for High Throughput Sequence Data, (2011).

[4] Bolger A.M., Lohse M., Usadel B. (2014). Trimmomatic: a flexible trimmer for Illumina sequence data. Bioinformatics.

[5] Langmead B., Salzberg S.L. (2012). Fast gapped-read alignment with Bowtie 2. Nat. Methods.

[6] Pruesse E., Quast C., Knittel K., Fuchs B.M., Ludwig W., Peplies J., Glöckner F.O. (2007). SILVA: a comprehensive online resource for quality checked and aligned ribosomal RNA sequence data compatible with ARB. Nucleic Acids Res..

[7] Quast C., Pruesse E., Yilmaz P., Gerken J., Schweer T., Yarza P., Peplies J., Glöckner F.O. (2013). The SILVA ribosomal RNA gene database project: improved data processing and web-based tools. Nucleic Acids Res..

[8] Fu L., Niu B., Zhu Z., Wu S., Li W. (2012). CD-HIT: accelerated for clustering the next-generation sequencing data. Bioinformatics.

[9] Li W., Godzik A. (2006). Cd-hit: a fast program for clustering and comparing large sets of protein or nucleotide sequences. Bioinformatics.

[10] Smith-Unna R., Boursnell C., Patro R., Hibberd J.M., Kelly S. (2016). TransRate: Reference-free quality assessment of de novo transcriptome assemblies. Genome Res..

[11] Simão F.A., Waterhouse R.M., Ioannidis P., Kriventseva E.V., Zdobnov E.M. (2015). BUSCO: assessing genome assembly and annotation completeness with single-copy orthologs. Bioinformatics.

[12] Waterhouse R.M., Seppey M., Simao F.A., Manni M., Ioannidis P., Klioutchnikov G., Kriventseva E.V., Zdobnov E.M. (2018). BUSCO applications from quality assessments to gene prediction and phylogenomics. Mol. Biol. Evol..

[13] Haas B.J., Papanicolaou A., Yassour M., Grabherr M., Blood P.D., Bowden J., Couger M.B., Eccles D., Li B., Lieber M., Macmanes M.D., Ott M., Orvis J., Pochet N., Strozzi F., Weeks N., Westerman R., William T., Dewey C.N., Henschel R., Leduc R.D., Friedman N., Regev A. (2013). De novo transcript sequence reconstruction from RNA-seq using the Trinity platform for reference generation and analysis. Nat. Protoc..

[14] Bryant D.M., Johnson K., DiTommaso T., Tickle T., Couger M.B., Payzin-Dogru D., Lee T.J., Leigh N.D., Kuo T.H., Davis F.G., Bateman J., Bryant S., Guzikowski A.R., Tsai S.L., Coyne S., Ye W.W., Freeman R.M., Peshkin L., Tabin C.J., Regev A., Haas B.J., Whited J.L. (2017). A tissue-mapped axolotl de novo transcriptome enables identification of limb regeneration factors. Cell Rep..

[15] Kanehisa M., Goto S. (2000). KEGG: Kyoto Encyclopedia of Genes and Genomes. Nucleic Acids Res..

[16] Powell S., Szklarczyk D., Trachana K., Roth A., Kuhn M., Muller J., Arnold R., Rattei T., Letunic I., Doerks T., Jensen L.J., Von Mering C., Bork P. (2012). eggNOG v3.0: orthologous groups covering 1133 organisms at 41 different taxonomic ranges. Nucleic Acids Res..

[17] Punta M., Coggill P.C., Eberhardt R.Y., Mistry J., Tate J., Boursnell C., Pang N., Forslund K., Ceric G., Clements J., Heger A., Holm L., Sonnhammer E.L.L., Eddy S.R., Bateman A., Finn R.D. (2012). The Pfam protein families database. Nucleic Acids Res..

[18] Bray N.L., Pimentel H., Melsted P., Pachter L. (2016). Near-optimal probabilistic RNA-seq quantification. Nat. Biotechnol..

